# Supplementary material for: Unveiling the Bioactive Potential of the Invasive Jellyfish Phyllorhiza punctata Through Integrative Transcriptomic and Proteomic Analyses
Source: Biomolecules. 2025 Aug 4;15(8):1121. doi: 10.3390/biom15081121 (PMC12383608; doi:10.3390/biom15081121)
Supplement: Supplementary file 1 [file biomolecules-15-01121-s001.zip › Suplementary Material/FileS3/PEPFOLD4/XP_065072335_17676/clusters.html]

 PEP-FOLD clusters


Download archive of all models  here   

|  |  |  |
| --- | --- | --- |
| # | file | sOPEP |
| # cluster 1 - Size: 5 |  |  |
|  | XP\_065072335\_17676-model1 | -14.1034 |
|  | XP\_065072335\_17676-model1.1 | -10.4374 |
|  | XP\_065072335\_17676-model1.2 | -9.74924 |
|  | XP\_065072335\_17676-model1.3 | -7.4144 |
|  | XP\_065072335\_17676-model1.4 | -6.71045 |
| # cluster 2 - Size: 13 |  |  |
|  | XP\_065072335\_17676-model2 | -11.0913 |
|  | XP\_065072335\_17676-model2.1 | -10.7465 |
|  | XP\_065072335\_17676-model2.2 | -9.89171 |
|  | XP\_065072335\_17676-model2.3 | -9.33636 |
|  | XP\_065072335\_17676-model2.4 | -8.73303 |
|  | XP\_065072335\_17676-model2.5 | -8.64783 |
|  | XP\_065072335\_17676-model2.6 | -8.54818 |
|  | XP\_065072335\_17676-model2.7 | -8.10862 |
|  | XP\_065072335\_17676-model2.8 | -7.95334 |
|  | XP\_065072335\_17676-model2.9 | -7.83446 |
|  | XP\_065072335\_17676-model2.10 | -7.746 |
|  | XP\_065072335\_17676-model2.11 | -7.13206 |
|  | XP\_065072335\_17676-model2.12 | -6.85114 |
| # cluster 3 - Size: 8 |  |  |
|  | XP\_065072335\_17676-model3 | -10.2622 |
|  | XP\_065072335\_17676-model3.1 | -9.1016 |
|  | XP\_065072335\_17676-model3.2 | -8.54258 |
|  | XP\_065072335\_17676-model3.3 | -8.3672 |
|  | XP\_065072335\_17676-model3.4 | -8.2319 |
|  | XP\_065072335\_17676-model3.5 | -7.94653 |
|  | XP\_065072335\_17676-model3.6 | -6.76991 |
|  | XP\_065072335\_17676-model3.7 | -6.61746 |
| # cluster 4 - Size: 5 |  |  |
|  | XP\_065072335\_17676-model4 | -9.21453 |
|  | XP\_065072335\_17676-model4.1 | -9.17985 |
|  | XP\_065072335\_17676-model4.2 | -8.01924 |
|  | XP\_065072335\_17676-model4.3 | -7.4511 |
|  | XP\_065072335\_17676-model4.4 | -6.7783 |
| # cluster 5 - Size: 23 |  |  |
|  | XP\_065072335\_17676-model5 | -8.90851 |
|  | XP\_065072335\_17676-model5.1 | -8.85305 |
|  | XP\_065072335\_17676-model5.2 | -8.81012 |
|  | XP\_065072335\_17676-model5.3 | -8.80695 |
|  | XP\_065072335\_17676-model5.4 | -8.61781 |
|  | XP\_065072335\_17676-model5.5 | -8.60653 |
|  | XP\_065072335\_17676-model5.6 | -8.58634 |
|  | XP\_065072335\_17676-model5.7 | -8.55644 |
|  | XP\_065072335\_17676-model5.8 | -8.48298 |
|  | XP\_065072335\_17676-model5.9 | -8.46541 |
|  | XP\_065072335\_17676-model5.10 | -8.41956 |
|  | XP\_065072335\_17676-model5.11 | -8.1443 |
|  | XP\_065072335\_17676-model5.12 | -7.98587 |
|  | XP\_065072335\_17676-model5.13 | -7.77506 |
|  | XP\_065072335\_17676-model5.14 | -7.68287 |
|  | XP\_065072335\_17676-model5.15 | -7.67768 |
|  | XP\_065072335\_17676-model5.16 | -7.65047 |
|  | XP\_065072335\_17676-model5.17 | -7.49091 |
|  | XP\_065072335\_17676-model5.18 | -7.29281 |
|  | XP\_065072335\_17676-model5.19 | -7.14474 |
|  | XP\_065072335\_17676-model5.20 | -7.0461 |
|  | XP\_065072335\_17676-model5.21 | -7.03794 |
|  | XP\_065072335\_17676-model5.22 | -7.01796 |
| # cluster 6 - Size: 8 |  |  |
|  | XP\_065072335\_17676-model6 | -8.71125 |
|  | XP\_065072335\_17676-model6.1 | -8.64361 |
|  | XP\_065072335\_17676-model6.2 | -8.56558 |
|  | XP\_065072335\_17676-model6.3 | -8.48892 |
|  | XP\_065072335\_17676-model6.4 | -8.398 |
|  | XP\_065072335\_17676-model6.5 | -8.17806 |
|  | XP\_065072335\_17676-model6.6 | -7.8901 |
|  | XP\_065072335\_17676-model6.7 | -7.08458 |
| # cluster 7 - Size: 2 |  |  |
|  | XP\_065072335\_17676-model7 | -8.68495 |
|  | XP\_065072335\_17676-model7.1 | -8.23216 |
| # cluster 8 - Size: 8 |  |  |
|  | XP\_065072335\_17676-model8 | -8.63232 |
|  | XP\_065072335\_17676-model8.1 | -8.12006 |
|  | XP\_065072335\_17676-model8.2 | -7.93058 |
|  | XP\_065072335\_17676-model8.3 | -7.90381 |
|  | XP\_065072335\_17676-model8.4 | -7.80155 |
|  | XP\_065072335\_17676-model8.5 | -7.59125 |
|  | XP\_065072335\_17676-model8.6 | -6.78268 |
|  | XP\_065072335\_17676-model8.7 | -6.55788 |
| # cluster 9 - Size: 8 |  |  |
|  | XP\_065072335\_17676-model9 | -8.40435 |
|  | XP\_065072335\_17676-model9.1 | -8.3912 |
|  | XP\_065072335\_17676-model9.2 | -8.15636 |
|  | XP\_065072335\_17676-model9.3 | -7.21192 |
|  | XP\_065072335\_17676-model9.4 | -7.09223 |
|  | XP\_065072335\_17676-model9.5 | -6.96019 |
|  | XP\_065072335\_17676-model9.6 | -6.76184 |
|  | XP\_065072335\_17676-model9.7 | -6.63606 |
| # cluster 10 - Size: 1 |  |  |
|  | XP\_065072335\_17676-model10 | -8.40297 |
| # cluster 11 - Size: 5 |  |  |
|  | XP\_065072335\_17676-model11 | -8.26349 |
|  | XP\_065072335\_17676-model11.1 | -8.09029 |
|  | XP\_065072335\_17676-model11.2 | -7.76321 |
|  | XP\_065072335\_17676-model11.3 | -7.50404 |
|  | XP\_065072335\_17676-model11.4 | -7.23516 |
| # cluster 12 - Size: 2 |  |  |
|  | XP\_065072335\_17676-model12 | -8.01827 |
|  | XP\_065072335\_17676-model12.1 | -7.26499 |
| # cluster 13 - Size: 9 |  |  |
|  | XP\_065072335\_17676-model13 | -8.00576 |
|  | XP\_065072335\_17676-model13.1 | -7.99424 |
|  | XP\_065072335\_17676-model13.2 | -7.97763 |
|  | XP\_065072335\_17676-model13.3 | -7.66371 |
|  | XP\_065072335\_17676-model13.4 | -7.63644 |
|  | XP\_065072335\_17676-model13.5 | -7.58382 |
|  | XP\_065072335\_17676-model13.6 | -7.29156 |
|  | XP\_065072335\_17676-model13.7 | -6.93231 |
|  | XP\_065072335\_17676-model13.8 | -6.84737 |
| # cluster 14 - Size: 2 |  |  |
|  | XP\_065072335\_17676-model14 | -7.77669 |
|  | XP\_065072335\_17676-model14.1 | -7.49336 |
| # cluster 15 - Size: 1 |  |  |
|  | XP\_065072335\_17676-model15 | -6.48394 |
|  |  |  |
